# The diagnostic value of convolutional neural networks in thyroid cancer detection using ultrasound images

**DOI:** 10.3389/fonc.2025.1534228

**Published:** 2025-05-08

**Authors:** Pei Zhang, Qijian Xu, Feng Jiang

**Affiliations:** Department of Ultrasound Medicine, The First Affiliated Hospital of Wannan Medical College, Wuhu, China

**Keywords:** ultrasound elastography, thyroid cancer prediction model, convolutional neural network, sonogram, machine learning

## Abstract

**Objective:**

To extract and analyze the image features of two-dimensional ultrasound images and elastic images of four thyroid nodules by radiomics, and then further convolution processing to construct a prediction model for thyroid cancer. The purpose of this study was to explore the diagnostic efficacy of the model.

**Methods:**

In this study, 199 cases of thyroid nodules were collected from August 2023 to July 2024, and all thyroid nodules had B-ultrasound-guided fine needle aspiration biopsy (FNA) pathological results/postoperative pathological results, including 79 cases of benign nodules and 120 cases of malignant nodules. In this study, four thyroid cancer prediction models were constructed and compared, including convolutional neural network (CNN), gradient boosting (GB), logistic regression (LR), and ultrasound and clinical feature models. In addition, the clinical feature model was constructed by using the clinical information of patients and ultrasound image features, and the predictive performance of four thyroid cancer models was evaluated and compared. The area under the receiver operating characteristic curve (AUC), accuracy, specificity, and sensitivity were used to validate the predictive power of the model. Finally, we used the Delong test to compare whether there was a significant difference in AUC between the four models.

**Results:**

The CNN model performed well in the Area Under the Curve (AUC) and ACC (Accuracy) indicators, reaching 0.853 and 0.85, respectively, which were significantly better than the Gradient Boosting, Logistics regression and clinical characteristics models. The AUC, ACC, SPE, and SEN of the Gradient Boosting model were 0.653, 0.67, 0.709, and 0.63, respectively, the Logistics regression model was 0.701, 0.71, 0.6, and 0.714, and the clinical characteristic model was 0.663, 0.69, 0.708, and 0.57, respectively. The outstanding performance of CNN highlights its potential in the field of image recognition.

**Summary:**

CNN model has shown strong predictive ability in ultrasound image analysis of suspicious thyroid nodules, which not only provides a powerful auxiliary diagnostic tool for clinicians, but also provides new directions and possibilities for future medical image analysis research.

## Background

Thyroid cancer is one of the most common endocrine cancers by far. With the fast-paced social life, the incidence of thyroid malignancy is increasing every year, according to global epidemiological data (1–4). The thyroid gland is one of the important endocrine organs of the human body, and there are many ways to examine the thyroid gland, such as physical examination, blood examination, CT, MRI, B-ultrasound, thyroid scintigraphy, and ultrasound-guided thyroid fine needle biopsy (5–7). But after comparison, ultrasound is now the most widely used means to detect and diagnose thyroid cancer (8–10). Ultrasonography is a safe, convenient, non-invasive, and reproducible diagnostic technique that can observe thyroid echo changes, accurately locate thyroid nodules, identify echo signatures within thyroid nodules, and discover annular punctate blood flow signals within nodules, allowing small lesions to be detected and blood flow within them assessed (11–15).

Elastography is an ultrasound technique for evaluating the stiffness of nodules (16–19). STE is a newly developed shear wave elastography technology combined with ultra-wide beam tracking technology, which can display shear wave elastic images in real time, which can not only detect the edge hardness of the nodule, but also detect the internal hardness (15, 20, 21). Although ultrasound is the mainstay of thyroid nodule evaluation, there is inter-observer variability in the diagnosis of C-TIRADS class 4 nodules, particularly in the differentiation of ‘indeterminate’ nodules (4a-4c). Most of the existing computer-aided diagnostic systems are based on B-ultrasound features or operation-dependent SWE technology, which has limited reproducibility. In this study, strain elastography (STE), a more stable technique than SWE, was used in combination with CNN radiomics methods to solve these problems.

Ultrasound radiomics can extract a large number of quantitative imaging features from ultrasound images, use artificial intelligence and other methods to combine ultrasound images with pathological histology, genetics or proteomics data of diseases, and provide additional information beyond what can be detected by conventional ultrasound, so as to improve the accuracy of ultrasound diagnosis (9, 15, 22). It is capable of extracting high-throughput features from ultrasound images, such as shape features, first-order grayscale histogram features, second-order and higher-order texture features, and filtering and transform-based features such as wavelet features (18, 22, 23). These features can reflect the heterogeneity within tumors and provide useful information for improving the diagnostic efficiency of tumors (10, 12, 21).

Clinically, ultrasound diagnosis is used as a screening technique, and after a suspicious malignant nodule is diagnosed, a fine-needle aspiration biopsy is usually performed to confirm the diagnosis (24, 25). Although ultrasound (US) and ultrasound elastography (STE) have been maturely applied to the diagnosis of various thyroid nodules, there are still some misdiagnoses and needle biopsies. US imaging requires specialized competence and hands-on experience because it is a handheld imaging modality that has not yet been standardized like other imaging modalities. Automatic identification of anatomical structures minimizes the dependence on subjective judgment of the examiner. Therefore, the application of new technologies such as machine learning algorithms in clinical routine can help to further improve the diagnosis of suspicious thyroid nodules and help avoid some unnecessary needle biopsies. Therefore, this study mainly investigated the accuracy of convolutional neural network combined with ultrasound technology in diagnosing suspected thyroid cases, and explored whether the accuracy can be further optimized to reduce unnecessary needle biopsy (26, 27, 28).

## Materials and methods

### Dataset

This retrospective study was approved by the Ethics Committee of our hospital and no written informed permission is required. A total of 199 patients with thyroid nodules diagnosed with C-TIRADS category 4 by ultrasound examination in our hospital from August 2023 to July 2024 and who underwent surgical treatment or B-ultrasound-guided fine needle aspiration biopsy with confirmed pathological results, including 65 males and 134 females. Inclusion criteria: (1) C-TIRADS score of 4 categories in routine ultrasonography without any clinical treatment; (2) All patients in this study had pathological results of B-ultrasound-guided fine needle aspiration biopsy/postoperative pathological results; (3) All patients underwent routine color Doppler ultrasound and ultrasound elastography before surgery, and the image data and clinical data were complete. Exclusion Criteria: (1) Poor image quality, the lump is too large, beyond the reach of the probe; (2) Those who have undergone radiotherapy and chemotherapy; (3) Patients with excessive internal calcification of the nodule accompanied by severe posterior sound and shadow.

**Figure 1:**
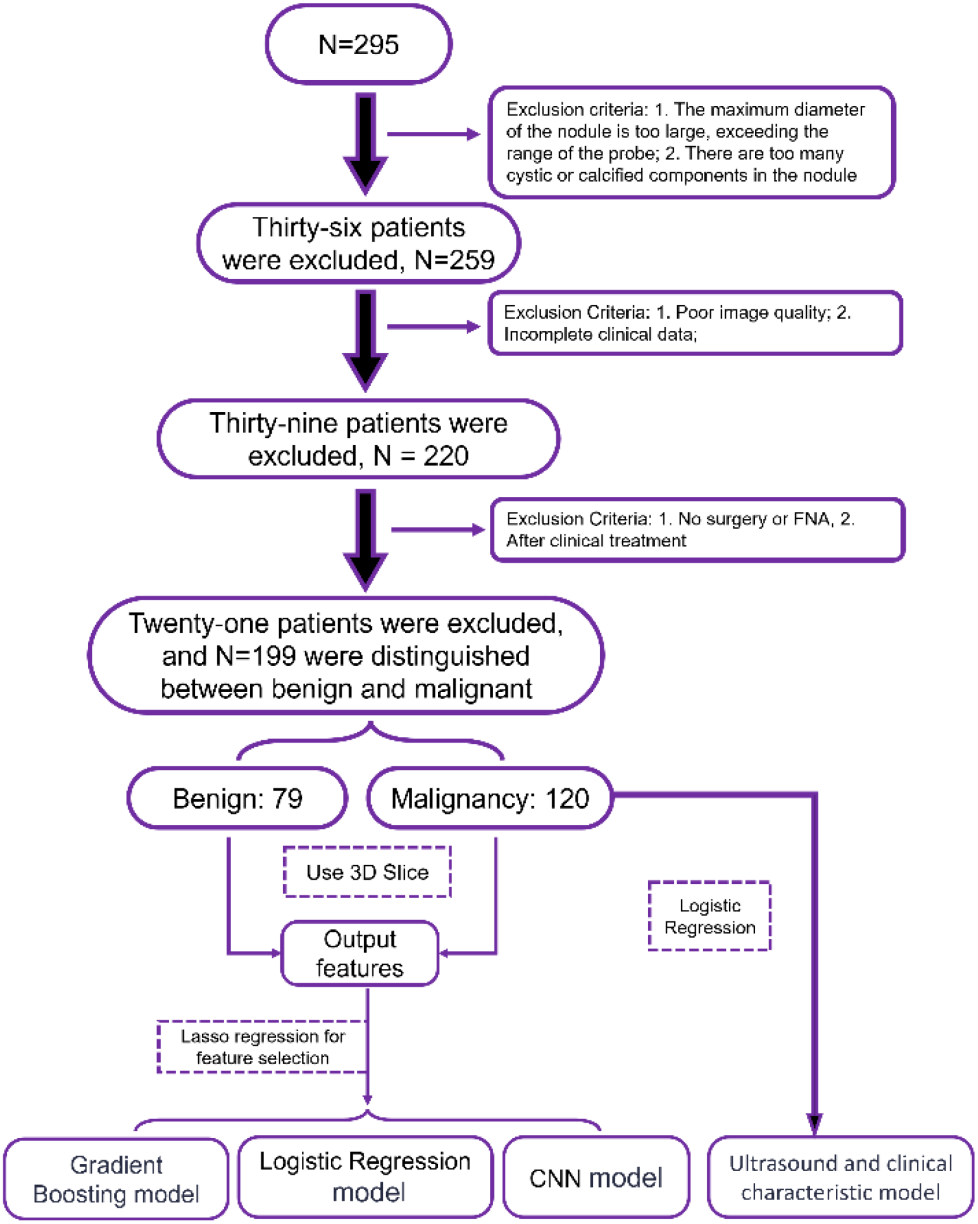
Schematic diagram of the collection process of patients with thyroid nodules.

We initially included 295 patients. However, in the course of the study, we encountered some situations that needed to be ruled out. First, 36 patients had nodules that exceeded the scope of our probes or had too many cystic or calcified components inside the nodules, which affected our accurate assessment of the nodules. Subsequently, 39 patients were excluded due to poor image quality or incomplete clinical data. Finally, there were 21 patients who did not have surgery or fine-needle aspiration biopsy (FNA) or who had already received clinical treatment, which also did not meet our criteria. After these screenings, we were finally left with 199 patients, including 79 benign nodules and 120 malignant nodules. These data provide us with a more precise sample population for subsequent analysis and research. The specific process is shown in the structural flow diagram of **Figure 1**.

### Image acquisition equipment and inspection methods

The ultrasound examination was performed by a sonographer with ten years of work experience, using a domestically produced Mindray Resona 7T ultrasound diagnosis, equipped with an L14–5 high-frequency linear array probe (its frequency range is 5-14MHz), with an STE mode inside. The patient was instructed to take a supine position, remove neck jewelry, fully expose the neck, apply an appropriate amount of couplant, and perform routine ultrasound examinations on the patient’s thyroid gland one by one in order, and ask the patient to avoid shaking, swallowing, coughing, etc. as much as possible during the examination. Adjust the instrument reasonably to obtain a clear image of the nodule and store it in the background of the instrument. After obtaining the target nodule through routine ultrasound, the maximum longitudinal section of the nodule was selected, the instrument was switched to sonotouch elasticography (STE) mode, the nodule was placed in the center of the area of interest, the patient was instructed to hold his breath for three seconds, and when the image was stable, no obvious artifacts were present, and the elastic quality was the best (i.e., the M-STB Index reached 4 or 5 stars), the image was frozen and stored in the background of the instrument.

### Data collection

The clinical characteristics of thyroid patients in our hospital were retrospectively collected in the follow-up and case system of our hospital, and the relevant ultrasound features were obtained from the acquired images. This information includes: patient gender, age, height, weight, smoking history, radiation history, history of automalignancy, family history of malignancy; Nodule location, size (<1 cm, >1 cm), morphology (regular, irregular), internal echo (hypoechoic, medium-high echoic), internal composition (solid, cystic), aspect ratio (>1, <1), margins (clear, blurred), relationship with the dorsal membrane (away from the dorsal membrane, close to the dorsal membrane, breaking through the dorsal membrane), calcification (coarse calcification, microcalcification, no calcification), Adler blood flow grading (grade 0, grade I, grade II, grade III).

### Feature extraction

When processing thyroid ultrasound image data, we implemented a series of pre-processing steps to ensure data consistency and comparability. First, all ultrasound images, including elastic and conventional ultrasound images, were standardized and normalized in a critical step designed to eliminate image differences due to different imaging devices or settings by uniformly adjusting the gray level of the images to a range of 0 to 1. Then, the Python programming language (version 3.7) was used in combination with its powerful open-source library Pyradiomics (version v3.1.0) to dig deeper into the radiomics features in these ultrasound images. This process covers not only the basic first-order features, but also the shape features, traditional texture features, and more complex higher-order texture features. The extraction of higher-order texture features relies in particular on advanced image processing techniques such as wavelet transform and Laplace Gaussian (LoG) filtering, which can reveal subtle structures and patterns in images that are not easily noticeable. In the end, we succeeded in extracting a total of 104 features from each thyroid ultrasound image, which comprehensively and exhaustively portrayed the multiple attributes of the image. These rich feature sets are then used as input data to construct a benign and malignant thyroid diagnostic model, aiming to accurately predict the nature (benign or malignant) of thyroid nodules through machine learning or deep learning algorithms.

### Model building

The process of processing 199 groups of ultrasound images of suspicious thyroid nodules and applying machine learning to classify them is a series of meticulous and systematic steps. First, participants were randomly divided into a training set and a test set in a 7:3 ratio, and the collected image data needed to undergo rigorous preprocessing to ensure image quality, including denoising, enhanced contrast, and standardized size. The PyRadiomics package was used to extract key quantitative features from the images, and the extracted features were normalized and standardized in the training set and the test set, respectively, and these features could reflect the morphological and textural information of the nodules. Subsequently, feature selection is performed to determine the subset of features that are most valuable to the classification task, reducing data dimensions and avoiding overfitting. The Minimum Absolute Shrinkage and Selection Operator Method (LASSO) was used to reduce the dimensionality of features, and the features were further selected, and the optimal tuning parameters were selected through 10-fold cross-validation, and the features with non-zero regression coefficients were selected from these candidate features.

In the analysis of ultrasound images of thyroid nodules, the extracted omics features were applied to three different machine learning models to evaluate their performance in the classification of benign and malignant thyroid nodules. Firstly, the scikit-learn library in Python 3.7 was used to construct a random forest (RF) and a logistic regression model. Random forest is an ensemble learning method that improves the accuracy and robustness of classification by constructing multiple decision trees and voting on them. Logistic regression is a simple linear model for dichotomous problems that predicts the probability of an event by estimating the maximum likelihood.

**Figure 2:**
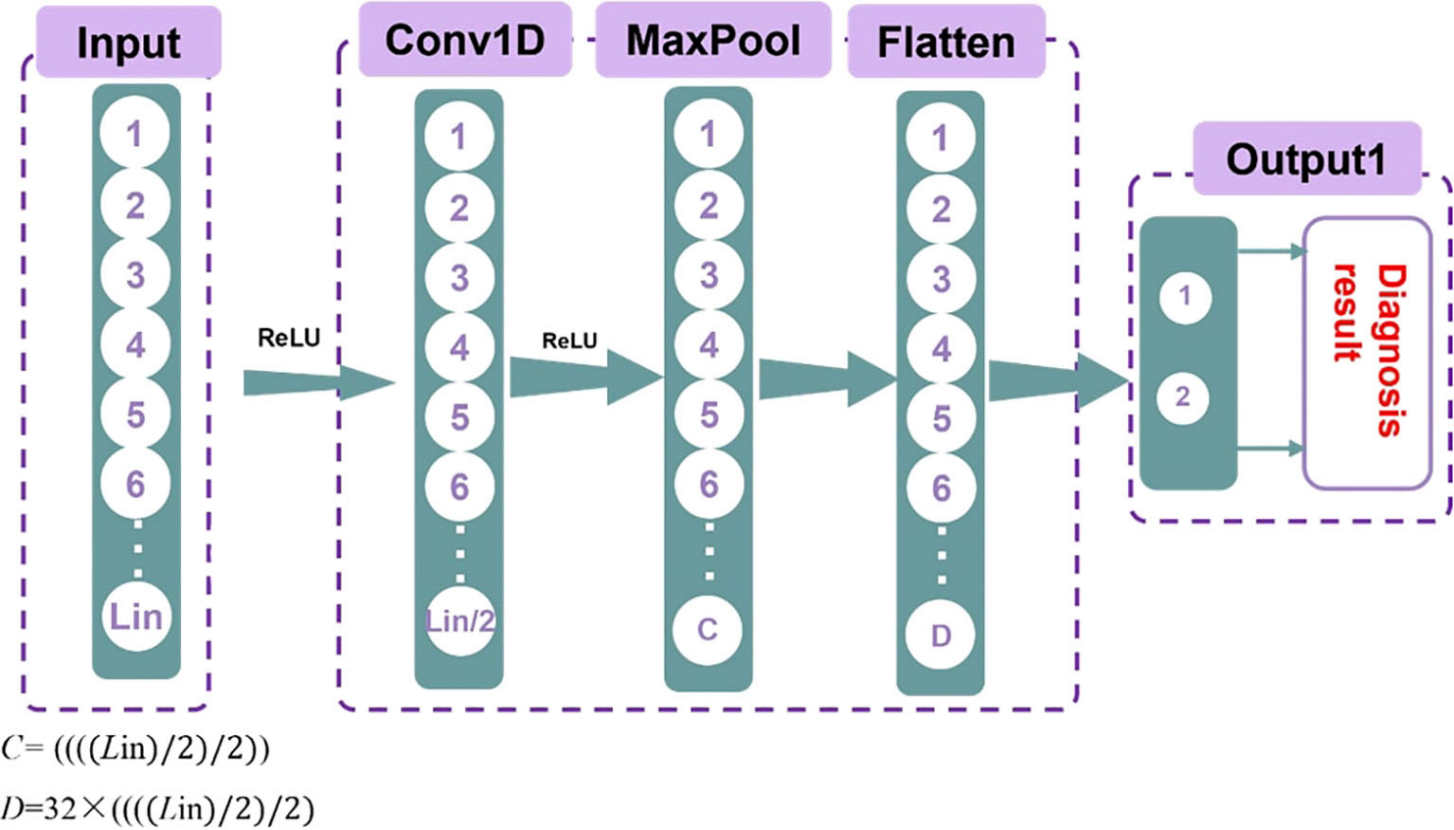
Schematic diagram of the CNN model structure.

Secondly, the convolutional neural network (CNN) model was built using the PyTorch package. One-dimensional CNNs are particularly effective for processing sequence data, as they can capture local features efficiently. Additionally, CNNs have a relatively small number of parameters, making them computationally efficient. This allows for faster training and inference, reducing the demand for computing resources. Moreover, CNNs are highly adaptable and can handle various types of ultrasound radiomics data. The structure of the model is illustrated in **Figure 2**.

In SPSS 27, a logistic regression model is constructed, and its statistical analysis capabilities are used to evaluate the predictive power of the model. After the model is built, the prediction results are displayed in tabular form, including the AUC values of each model on the training and test sets. The AUC value, i.e., the area under the receiver operating characteristic curve, is an important indicator to evaluate the performance of the classification model, which can reflect the ability of the model to distinguish between the two categories.

By comparing the AUC values of different models on the training set and the test set, the model with the best performance was selected. For the selected optimal model, the ROC curves of the training set and the validation set are plotted and summarized. Finally, AUC, accuracy (ACC) and confusion matrix were used to comprehensively evaluate the performance of the model.

## Outcome

### Clinical features

A total of 199 cases were included in this study, including 120 cases of malignant nodules and 79 cases of benign nodules. Among them, there were 65 males and 134 females, aged 21~83 years old, with an average age of 47.4 ± 13.6 years. There were statistically significant differences between the benign and malignant groups in the size, morphology, internal composition, aspect ratio, and family history of malignant tumors (P < 0.05), however, there were no significant differences between the benign and malignant groups in terms of age, height, weight, gender, smoking history, radiation history, menstrual status, mass location, margin, and the relationship between the mass and the dorsal membrane (P > 0.05), as shown in **Table 1**.

**Table 1:** Shows the distribution of chi-square clinical features.

Clinical and Ultrasound Characteristics	Malignant group (n=120)	Benign group (n=79)	χ^2^	P
Gender				
man	35 (63.4%)	20 (36.6%)	0.353	0.552
woman	85 (59.0%)	59 (41.0%)		
Menopause				
\	34 (63.0%)	20 (37.0%)	3.452	0.178
Not postmenopausal	48 (66.7%)	24 (33.3%)		
Postmenopausal	38 (52.1%)	35 (47.9%)		
Family history of malignancy				
Not	100 (56.8%)	76 (43.2%)	7.718	0.005*
Yes	20 (87.0%)	3 (13.0%)		
History of smoking				
Not	85 (58.2%)	61 (4.8%)	0.993	0.319
Yes	35 (66.0%)	18 (34.0%)		
History of radiology				
<3	45 (60.0%)	30 (40.0%)	0.005	0.946
>3	75 (60.5%)	49 (39.5%)		
History of malignancy				
Not	115 (61.2%)	73 (38.8%)	0.516	0.472
Yes	5 (45.5%)	6 (54.5%)		
Morphology				
Irregular	75 (67.6%)	36 (32.4%)	5.536	0.019*
Regular	45 (51.1%)	43 (48.9%)		
The site of the lump				
Isthmus	7 (53.8%)	6 (46.2%)	6.772	0.342
Upper right lobe	14 (82.4%)	3 (17.6%)		
Middle right lobe	18 (62.1%)	11 (37.9)		
Lower right lobe	30 (57.7%)	22 (42.3%)		
Upper left lobe	14 (73.7%)	5 (26.3%)		
Left lobe in the middle	11 (57.9%)	8 (42.1%)		
Lower left lobe	26 (52.0%)	24 (48.0%)		
Internal composition				
Cystic	5 (33.3%)	10 (66.7%)	4.929	0.026*
Solidity	115 (62.5%)	69 (37.5%)		
Relationship with the dorsal				
Stay away from the back membrane	46 (56.8%)	35 (43.2%)	1.514	0.469
Keep away from the back	59 (60.8%)	38 (39.2%)		
Breakthrough the back membrane	15 (71.4%)	6 (28.6%)		
Aspect ratio				
<1	58 (52.3%)	53 (47.7%)	6.794	0.009*
>1	62 (70.5%)	26 (29.5%)		
The size of the lump				
<1	93 (65.5%)	49 (34.5)	5.581	0.018*
>1	27 (47.4%)	30 (52.6%)		

*P<0.05, the difference is statistically significant.

### Clinical feature analysis and model establishment

**Figure 3:**
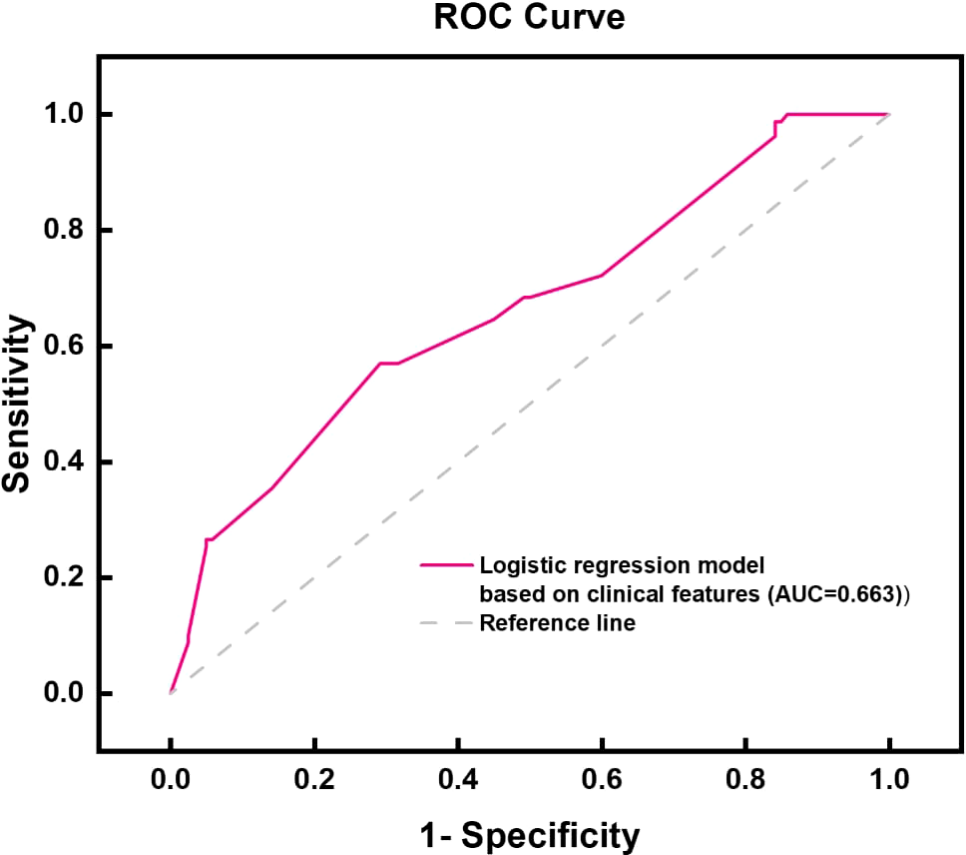
ROC curve chart based on the logistic regression model of clinical characteristic model.

By integrating the clinical features of thyroid cancer, such as tumor size, morphology, internal composition, aspect ratio, and family history of malignant tumors, and using logistic regression analysis, a thyroid benign and malignant classification model was successfully constructed, as shown in **Table 2**. This model can effectively classify and predict thyroid cancer based on these clinical features. The accuracy and predictive ability of the model were demonstrated through the ROC curve, as shown in **Figure 3**. The AUC value of the thyroid benign and malignant classification model based on clinical features can reach 0.663. This curve comprehensively considers the sensitivity and specificity of the model, and evaluates its diagnostic performance.

**Table 2:** Logistic regression analysis of thyroid ultrasound images.

Clinical and Ultrasound Characteristics	β	Standard deviation	P	OR	95% CI
Family history of malignancy	1.591	0.659	0.016	4.909	1.349~17.870
The size of the lump	-0.457	0.355	0.198	0.633	0.316~1.270
Morphology	-0.373	0.333	0.263	0.688	0.358~1.323
Internal composition	0.744	0.647	0.250	2.104	0.592~7.471
Aspect ratio	0.461	0.339	0.174	1.586	0.816~3.083
Constant	-1.672	0.803	0.037	0.188	

### Ultrasound imaging omics feature extraction

In the omics feature analysis of ultrasound medical images, I used machine learning, convolutional neural networks (CNN), and logistic regression methods to conduct in-depth mining and analysis of image data. Through LASSO feature dimensionality reduction technology, we have selected the most representative features from a large number of image features to construct an efficient classification model.

For the machine learning model, we ultimately selected five ultrasound imaging omics features that showed high discriminative ability in image analysis. They are original_firorder_Sinimum: minimum eigenvalue.

**Figure 4:**
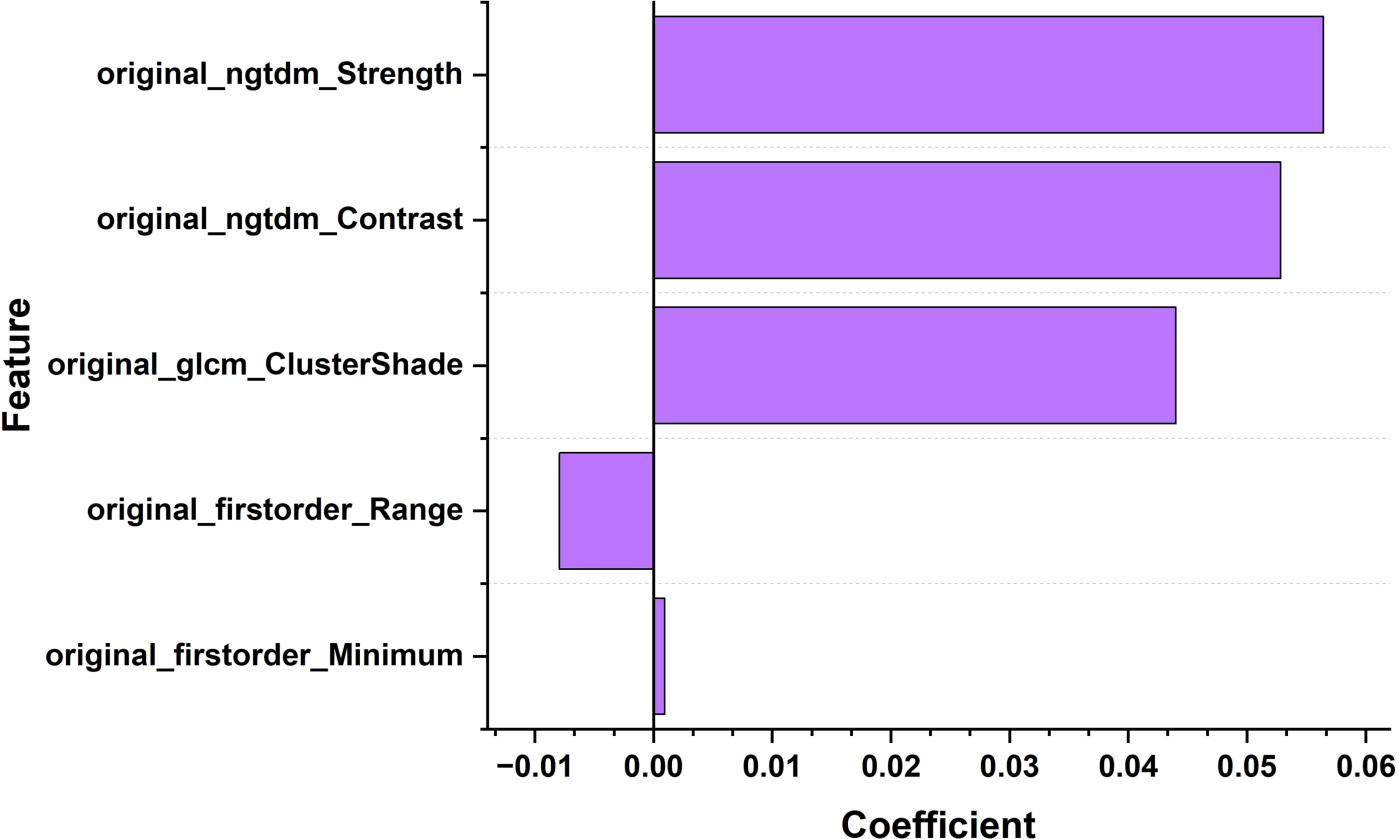
Bar chart of the correlation coefficients of ultrasound imaging biomarker features for thyroid nodules.

**Figure 5:**
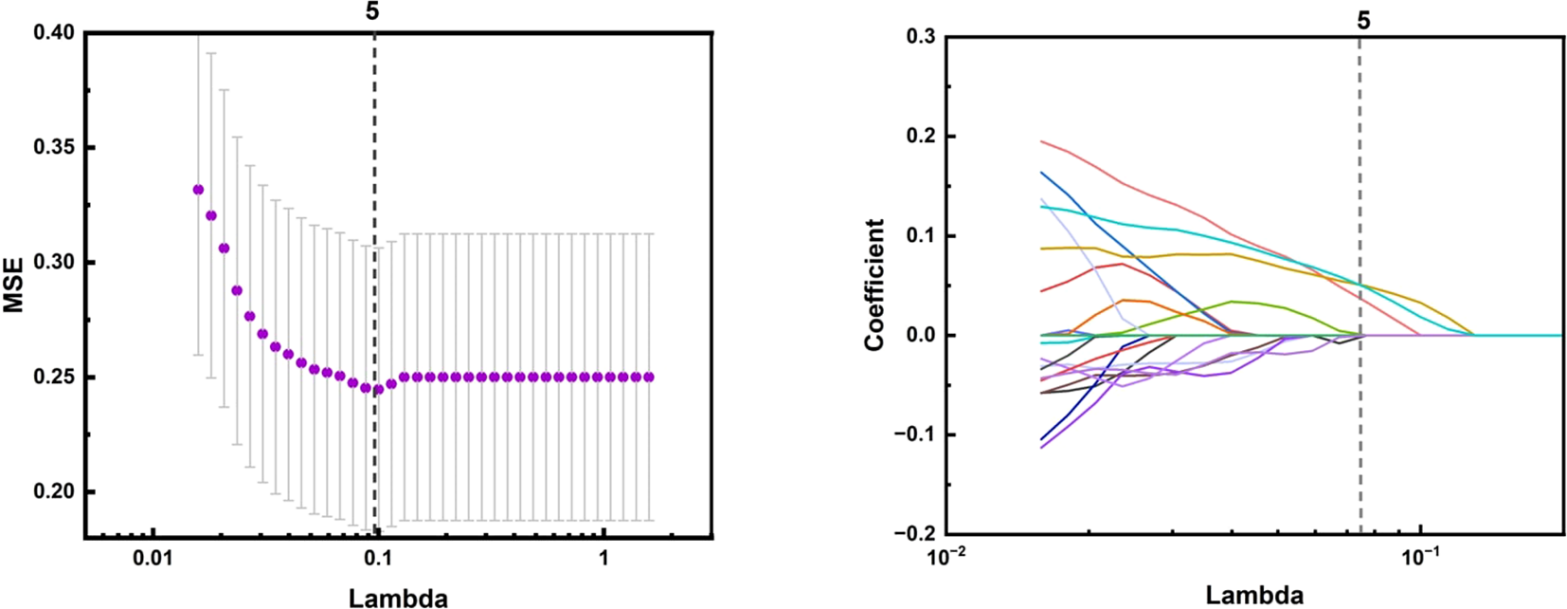
The LASSO algorithm for feature selection in ultrasound imaging-based radiomics of thyroid nodules: A feature selection map screened by LASSO algorithm.

Origina_firstorder_range: range of grayscale values; Original-glcm_ClusterShade: Cluster shadow, an indicator used to measure the skewness and uniformity of GLCM; Original-ngtdm_Cntrast: contrast; Original-ngtdm_Strength: Intensity, as shown in **Figures 4**, **5**, the minimum eigenvalue, grayscale value range, and correlation coefficient values of cluster shadows can be observed to reach 0.05643, 0.04401, and 0.05286, respectively. Among the five radiomics features, in the convolutional neural network model, we further refined the feature selection and selected 10 features, including the original first-order statistical features (such as minimum value, range, etc.), the features of the gray level co-occurrence matrix (GLCM) (such as Cluster Shade), and the features of the adjacent gray level tone difference matrix (NGTDM) (such as Contrast and Strength).

The selection of these features is based on their importance in image texture analysis. For example, first-order statistical features can reflect the grayscale distribution of the image, while GLCM and GLDM can reveal the texture information of the image, such as roughness, uniformity, and directionality. NGTDM can provide additional information about grayscale changes in images.

In the convolutional neural network model, in addition to the above features, there are also percentile based statistical features such as 10th percentile, 90th percentile, and maximum value, which can provide more details about the grayscale distribution of the image. In addition, it also includes variance features that reflect local dependencies and changes in the image, such as the Dependence Variation of GLDM and the Gray Level Non Uniformity of GLRLM.

By fusing these features, convolutional neural networks can capture both micro and macro features of images, thereby improving the accuracy of the model in classifying benign and malignant thyroid nodules. The weight graph analysis shows that specific features such as wavelet-LHH_glcmSizeShade_CC and log-sigma-3-0-mm3D_glcmContrast MLO have significant weights in the model, indicating that they play a key role in classification decision-making.

### Model effectiveness evaluation

**Figure 6:**
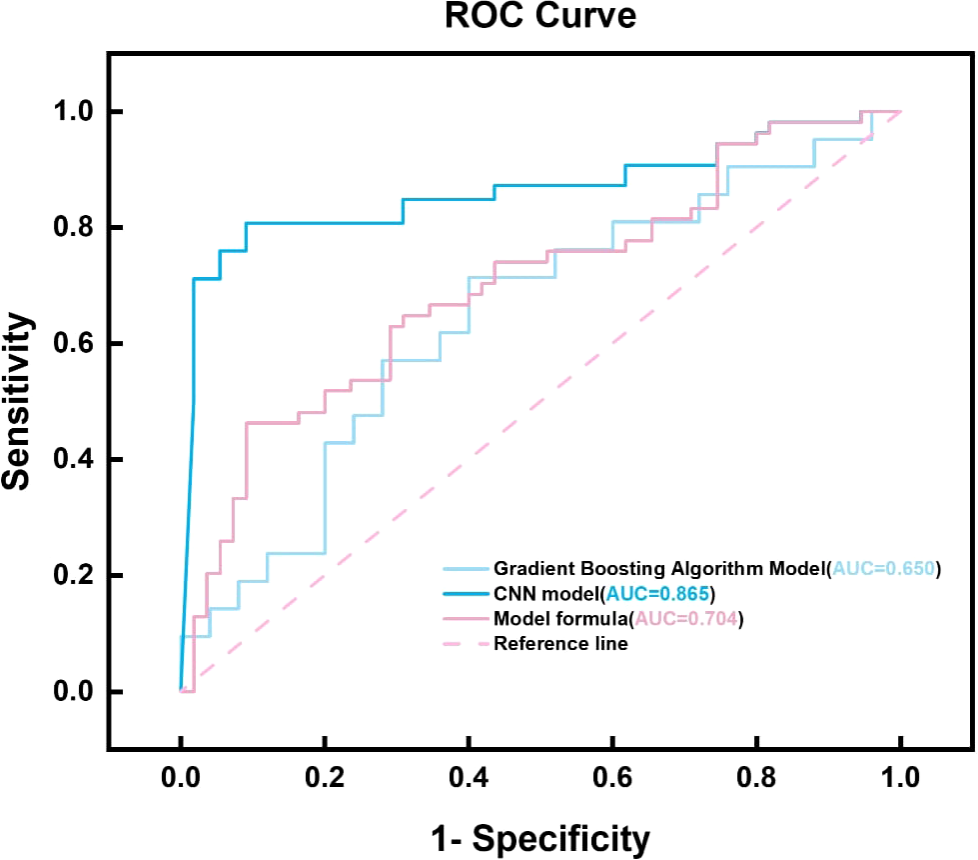
ROC plots of four model.

**Figure 7:**
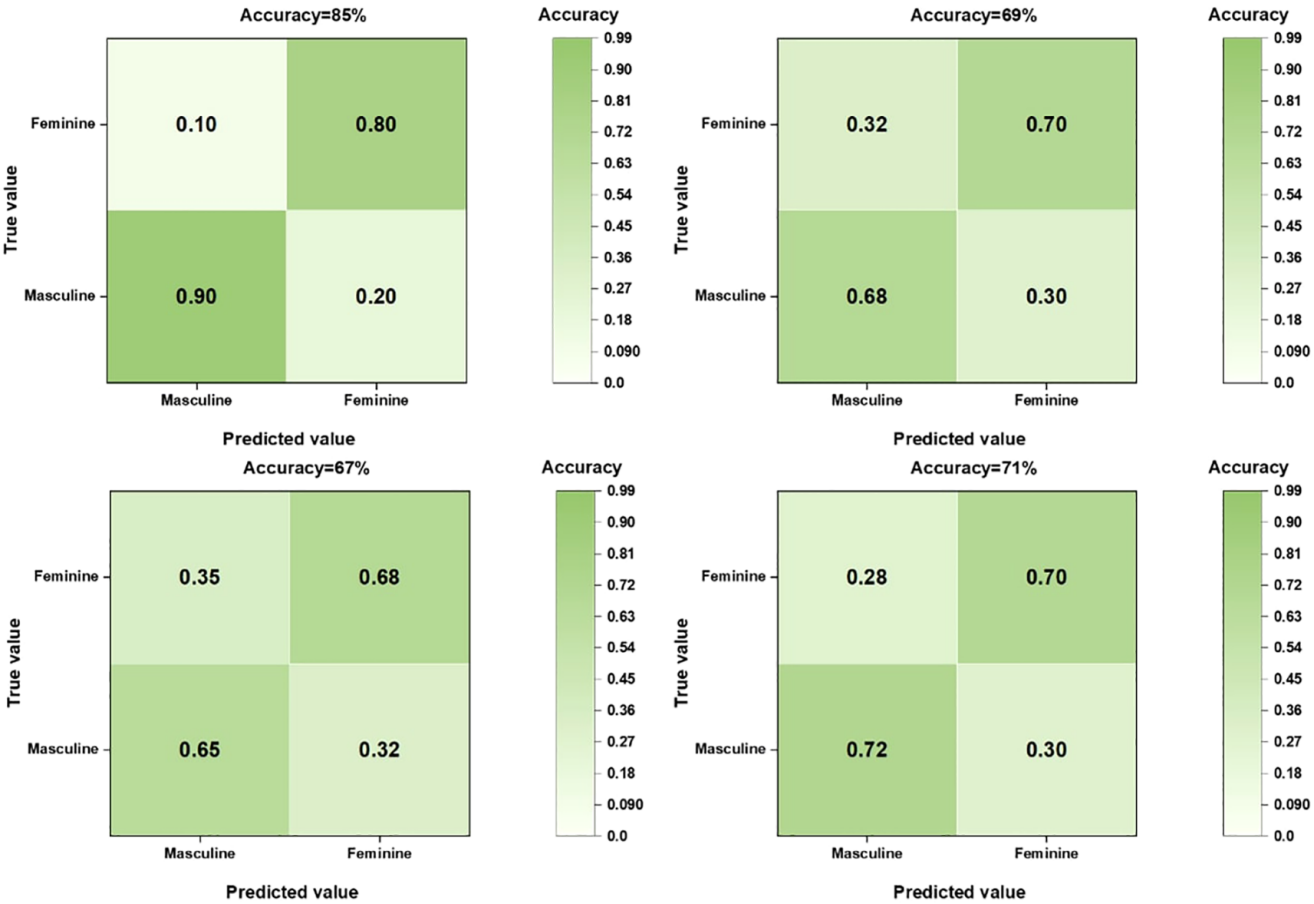
Confusion matrix of four models for predicting benign or malignant thyroid cancer.

When comparing the diagnostic performance of four model classification algorithms for benign and malignant thyroid cancer, we found that the CNN classification model had the best predictive performance on the test set, as shown in **Table 3**, with the highest AUC value of 0.853 (95% confidence interval: 0.773-0.933), and demonstrated high accuracy (ACC) of 0.85, specificity (SPE) of 0.909, and sensitivity (SEN) of 0.807 on the training set. The Gradient Boosting classification model performs the worst among all models, with an AUC value of 0.653 (95% confidence interval: 0.504-0.802), accuracy of 0.67, specificity of 0.709, and sensitivity of 0.63. The AUC value of the Logistics Returns classification model is 0.701 (95% confidence interval: 0.583-0.819), with an accuracy of 0.71, specificity of 0.6, and sensitivity of 0.714. The AUC value of the Clinical Features classification model is 0.663 (95% confidence interval: 0.544-0.782), with an accuracy of 0.69, specificity of 0.708, and sensitivity of 0.57. Overall, the CNN classifier performs the best among all models, with the highest AUC value and overall performance. As shown in **Figure 6**, the performance of Gradient Boosting classifiers is generally poor, while Logistics Returns and Clinical Features classifiers, although performing fairly well in some metrics, are inferior to CNN classification models in overall performance. Therefore, based on its comprehensive performance, we choose CNN as the preferred diagnostic algorithm for benign and malignant thyroid cancer. **Figure 7** shows the classification results of the model constructed by the CNN classifier. The DeLong test showed that there was a significant difference in AUC values between Gradient Boosting and CNN (p=0.035), as well as between CNN and Clinical Features and Logistics Returns (p=0.015 and p=0.00015), as shown in **Table 4**.

**Table 3:** Predictive efficacy of CNN, gradient boosting model, logistic regression model, and clinical feature model.

Model classification	AUC	ACC	SPE	SEN
CNN	0.853	0.85	0.909	0.807
Gradient Boosting	0.653	0.67	0.709	0.63
Logistics returns	0.701	0.71	0.6	0.714
Clinical featurs	0.663	0.69	0.708	0.57

**Table 4:** Delong test of gradient boosting model, logistic regression model, and clinical feature model relative to CNN model.

Model classification	Z	P	AUC differences	Standard deviation	95% CI
Gradient Boosting	2.107	0.035*	0.189	0.090	0.013~0.366
Logistics returns	2.444	0.015*	0.152	0.062	0.030~0.274
Clinical features	3.509	1.05×10^-4^*	0.190	0.054	0.084~0.296

*P<0.05, the difference is statistically significant.

## Discuss

Convolutional neural networks (CNNs) have shown significant advantages in the field of medical image analysis, especially in the classification of benign and malignant thyroid nodules, compared to other traditional models such as gradient boosting models, logistic regression models, and models that rely solely on clinical information. CNN can automatically learn complex feature representations from image data through its deep learning architecture, eliminating the tedious manual feature engineering, which is particularly valuable in medical image analysis.

In this study, by comparing and analyzing the predictive value of CNN with other models, the performance of each model can be comprehensively evaluated, including the comparison of key indicators such as accuracy, sensitivity, specificity, and AUC value. Through the predictive performance tables of CNN, gradient boosting model, logistic regression model, and clinical feature model, as well as the confusion matrix of the four models for predicting benign and malignant thyroid cancer, we can intuitively see the high AUC and ACC values of the CNN model. The performance of the CNN model on the training set is significantly better than other models. Although its specificity is slightly lower than sensitivity, it has the highest AUC and ACC values, as well as the highest specificity and sensitivity, and performs the best in the test set. The performance of Gradient Boosting model and Logistics Returns model is similar, but they each have advantages in specificity and sensitivity. The Clinical Features model needs further improvement as it performs poorly on all evaluation metrics.

Delong test is a commonly used statistical method to evaluate whether there are significant differences in the area under the operating characteristic curve (AUC) of subjects in different diagnostic models when comparing model performance. In this study, we used the Delong test to compare the Delong test table of three different models, namely the gradient boosting model, logistic regression model, and clinical feature model, relative to the CNN model. The results showed that the CNN model performed well in predicting the malignancy of thyroid cancer, with significantly higher AUC values than the other two models and the prediction model that only used clinical features. This significant difference is statistically significant because the P-values are all less than 0.05, indicating that the predictive performance of the CNN model is significantly better than other models in statistics. In contrast, although the Gradient Boosting model and Logistics regression model also have some predictive ability, they have not reached the level of CNN models in terms of AUC values.

In summary, CNN models have high accuracy and predictive ability in distinguishing benign and malignant thyroid nodules. Moreover, CNN models have significant advantages in processing complex image data, especially in the field of medical image analysis, such as benign and malignant classification of thyroid nodules. This may be because CNN can automatically learn complex feature representations from images without manually designing feature extraction algorithms, thereby improving the model’s generalization ability and prediction accuracy.

However, this study has certain limitations: 1. As a retrospective study, it is difficult to avoid certain selection biases; 2. This study is a single center, small sample size study, and further sample size expansion and multi center validation of the model are needed in the future; 3. Manually delineating ROI not only has certain differences, but also increases the required time. In the future, we will explore deep learning as an automatic segmentation method to replace manual delineation.

The CNN model can automatically learn the hierarchical features of images through its multi-layer structure, which is particularly prominent when compared with other traditional models such as gradient boosting models, logistic regression models, and models that rely solely on clinical information. This article specifically applies the CNN architecture to ultrasound radiomics, and greatly improves the accuracy of thyroid cancer diagnosis through radiomics analysis of two-dimensional ultrasound images and ultrasound elastography of thyroid nodules. This was relatively rare in previous studies.

In addition, research progress in the field of medical image analysis also indicates that deep learning models need to consider the specificity of medical image data when processing it, such as high inter class similarity, limited data volume, label noise, and other issues. In order to improve the performance of the model, researchers have adopted various strategies, including transfer learning, unsupervised learning, semi supervised learning, and self supervised learning. These methods help improve the performance and generalization ability of the model with limited labeled data.

Using CNNs for thyroid cancer detection has great potential to improve clinical practice. These models can analyze ultrasound images accurately, helping doctors make better decisions. For example, CNNs could be used as a first step to identify suspicious nodules, allowing doctors to focus on more complex cases. This could reduce diagnostic errors and make the process faster and more efficient.

However, there are some challenges. The model’s performance depends on the quality and size of the dataset used for training. If the dataset is too small or lacks diversity, the model might not work well for all patients. Future research should focus on using larger, more diverse datasets and improving the model’s ability to handle different types of ultrasound images.

Another issue is that CNNs can be like a “black box,” making it hard to understand how they make decisions. Developing methods to explain the model’s predictions could help doctors trust and use it more effectively.

In the future, combining ultrasound images with other patient data, like medical history or lab results, could make the model even more accurate. Testing the model in real-world clinics will also be important to see how well it works in practice.

Overall, CNNs have the potential to transform thyroid cancer diagnosis, but more work is needed to address current limitations and ensure they can be used effectively in hospitals.

## Data Availability

The raw data supporting the conclusions of this article will be made available by the authors, without undue reservation.
